# Trends in Intra-Aortic Balloon Pump Technology: Patentometric Analysis

**DOI:** 10.2196/98093

**Published:** 2026-11-06

**Authors:** Huimin Shen, Jianchao Li, Xue Dong, Chunjing Tao

**Affiliations:** 1 Beijing Advanced Innovation Center for Biomedical Engineering, Beijing, China; 2 School of Engineering Medicine, Beihang University, No. 37 Xueyuan Road, Haidian District, Beijing, 100191, China

**Keywords:** mechanical circulatory support, hemodynamics, intra-aortic balloon pump, patent analysis, Equipment Design

## Abstract

**Background:**

The intra-aortic balloon pump (IABP) remains a commonly used temporary mechanical circulatory support device because it is relatively easy to implant, less invasive, and well established in clinical practice. Previous studies have focused primarily on the physiological mechanisms, indications, clinical outcomes, and complications of IABP, whereas its technological evolution and industrial research have not been systematically examined.

**Objective:**

This study aimed to characterize the global technological and industrial evolution of IABP; identify current research priorities and underdeveloped areas; and relate patent innovation to unresolved needs in hemodynamic support, vascular safety, counterpulsation accuracy, prolonged bridging, and patient transport.

**Methods:**

Patent documents related to IABP were retrieved from the PatSnap database using a combined search strategy based on keywords and International Patent Classification (IPC) codes. The search was limited to documents published by August 18, 2026. After screening and deduplication, simple patent families were used as the unit of analysis. Patentometric analysis and technology function matrix analysis were performed to characterize the technological evolution, geographical distribution, and major innovation directions of IABP technology.

**Results:**

A total of 1235 IABP-related patent families were identified. The United States, China, Japan, and Europe were the principal countries or regions of origin, together accounting for 89.1% (1100/1235) of the included simple patent families. China contributed 326 simple patent families, representing 26.4% of the global total, and showed particularly rapid growth over the past 5 years. IABP technology evolved through an embryonic stage (1968-1985), a steady development stage (1986-2012), and a rapid development stage beginning in 2013. Patent families were mainly classified as balloon components (n=552, 44.7%), catheters and accessories (n=416, 33.7%), and counterpulsation timing and control (n=161, 13%). The technology function matrix indicated that structural optimization and control precision are the current priorities in research and development, while balloon folding and compression, fiber-optic pressure sensing, and adaptive control algorithms have emerged as important hotspots.

**Conclusions:**

IABP innovation has focused on balloon catheter design, pressure sensing, and adaptive control to improve hemodynamic support and counterpulsation accuracy. However, safety and complication reduction remain underdeveloped, particularly for prolonged support and transport. Future systems should integrate durable, low-profile designs with sensing, adaptive timing, and failure monitoring and be evaluated in specific patient populations and clinical settings.

## Introduction

The intra-aortic balloon pump (IABP) is one of the most widely used devices for temporary percutaneous mechanical circulatory support (pMCS). During diastole, the balloon inflates to augment aortic diastolic pressure and coronary perfusion; immediately before systole, it rapidly deflates to reduce left ventricular afterload and myocardial oxygen demand [1]. Some studies have suggested that prophylactic preoperative IABP use may reduce perioperative myocardial ischemia and mortality in high-risk patients undergoing coronary artery bypass grafting [2,3]. However, IABP provides limited hemodynamic support, with substantial interindividual variability. Its survival benefit remains uncertain in some clinical settings [4,5], and vascular complications such as bleeding and limb ischemia persist [6]. Previous studies have focused primarily on the physiological mechanisms, indications, clinical outcomes, and complications of IABP, whereas its technological evolution, industrial research and development landscape, and translation into commercial products have not been systematically examined [7].

Recent advances in materials science, sensing technologies, and intelligent control have opened new avenues for optimizing IABP structure, monitoring device status, and controlling counterpulsation timing [7]. Patent documents contain information on device designs, control methods, and assignees, providing a valuable basis for examining technological evolution and the global research and development landscape [8,9].

Therefore, using simple patent families as the unit of analysis, this study applied patentometric and technology function matrix analyses to characterize the global technological evolution and research and development landscape of IABP. It also examined the alignment between patented innovation directions and clinical needs related to inadequate hemodynamic support, vascular complications, triggering errors, prolonged support, bridging therapy, and patient transport. Relevant scientific literature and commercially available products were further considered to identify current technological gaps and inform future research and product development.

## Methods

### Study Scope and Technical Framework

Figure 1 summarizes the operating principle and major components of an IABP system and was used to define the technical scope of the patent search, eligibility screening, and subsequent technology classification. For this study, IABP technology was considered to comprise the balloon; catheter and insertion accessories; drive and pneumatic systems; sensing and triggering components; and timing, monitoring, and safety control functions.

**Figure 1.**
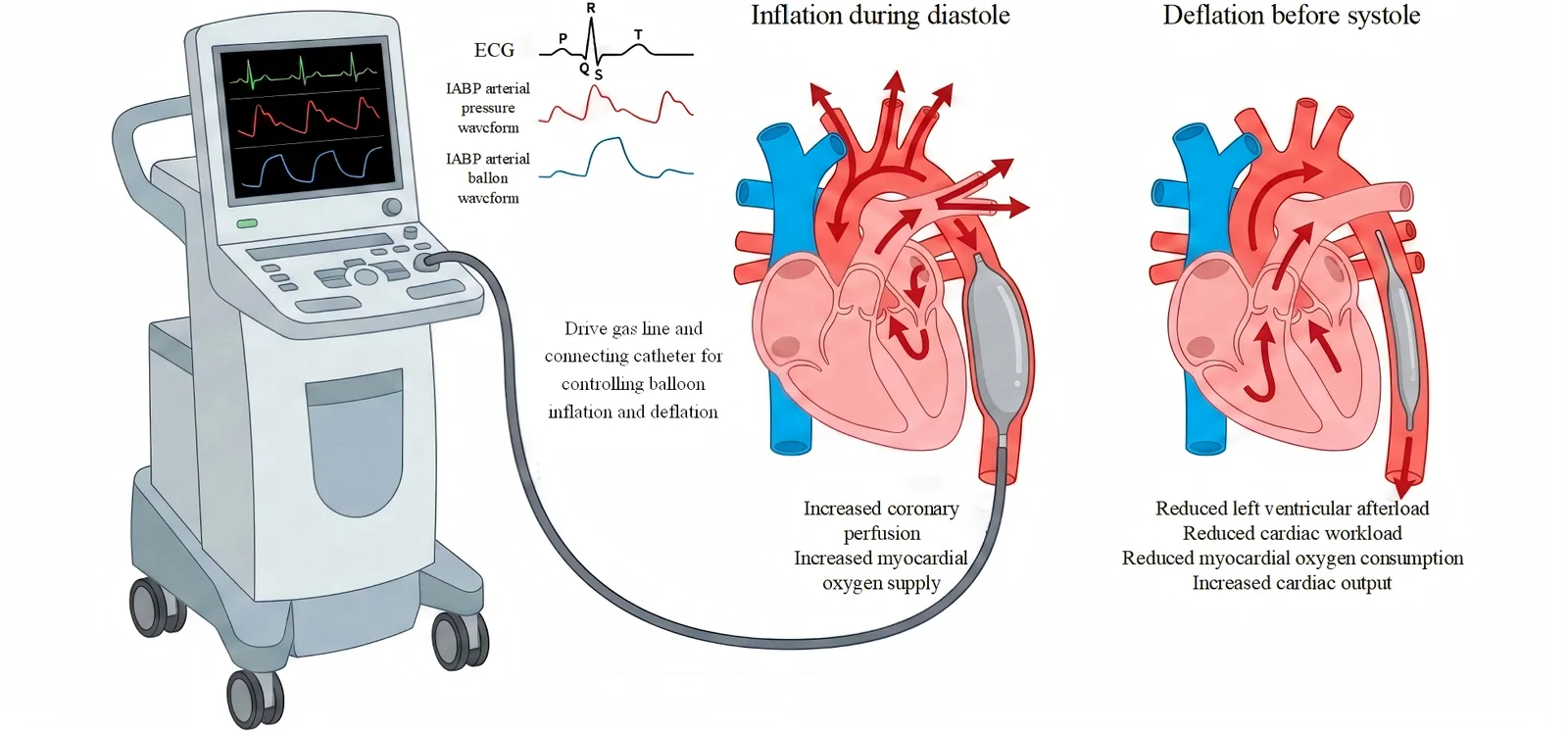
Schematic diagram of the working principle of intra-aortic balloon pump (IABP). ECG: electrocardiogram.

### Data Source and Search Strategy

The patent search was conducted in the PatSnap Analytics database (PatSnap Pte Ltd) on August 18, 2026. Patent records published on or before this date were included, with no lower date limit. The search covered all patent authorities, with no restrictions on jurisdiction, applicant, language, or legal status. Published patent applications, granted patents, and utility models were included, whereas design patents were excluded.

The search terms covered current and historical IABP terminology, phases of operation, drive and control components, and relevant International Patent Classification (IPC) codes [10]. The search fields were defined as follows: TACD_ALL searched titles, abstracts, claims, and descriptions; TAC_ALL searched titles, abstracts, and claims; and IPC searched IPC codes. The search modules were combined using OR and executed as a single query. The full search string is shown in Textbox 1.

Textbox 1. **Search string.**

(TACD_ALL:((“intra-aortic balloon” OR “intraaortic balloon” OR “intra aortic balloon” OR “intra-aortal balloon” OR “intraaortal balloon”) AND (pump OR pumping OR counterpulsation OR (counter pulsation)) AND ((diastolic (inflation OR augmentation)) OR (systolic (deflation OR unloading)) OR ECG OR electrocardiogram OR synchronization OR synchronized OR trigger OR driver OR console OR helium))

OR

TAC_ALL:(“phase-shift balloon pumping” OR “phase shift balloon pumping” OR “phase-shift balloon pump” OR “phase shift balloon pump” OR “intraaortic counterpulsator” OR “intra-aortic counterpulsator” OR (IABP (balloon OR pump)))

OR

TAC_ALL:(((aorta balloon) OR (aortic balloon)) AND (counterpulsation OR (counter pulsation)))

OR

TAC_ALL:((balloon deflation) AND counterpulsation)

OR

(IPC:(A61M60/139 OR A61M60/295 OR A61M60/841 OR A61M60/843 OR A61M1/10 OR A61M25/00 OR A61M25/01 OR A61M25/10 OR A61L29/00 OR A61M39/00 OR A61M60/865 OR A61M60/867 OR A61M60/497 OR A61M60/508 OR A61M60/515 OR A61M60/531 OR A61M60/569 OR A61B5/0215 OR A61B5/318)

AND

TAC_ALL:(((aorta balloon) AND counterpulsation) OR (IABP (balloon OR pump)))))

### Data Export and Patent Family Processing

The search results were collapsed at the PatSnap simple patent family level, and 1 representative record was exported for each family. The exported fields included publication number, application number, title, abstract, claims, applicant, inventor, publication date, filing date, earliest priority date and country, IPC codes, simple patent family ID, and family members.

In R (version 4.4.1; R Foundation for Statistical Computing), publication numbers were converted to uppercase and stripped of spaces and hyphens. Records with identical publication numbers were removed first, followed by a check for duplicate simple patent family IDs. If multiple records remained for the same family, the record with the most complete English title, abstract, and relevant independent claims was retained. Where completeness was equivalent, the record with the earliest publication date was selected. Application numbers were not used as a separate basis for deduplication.

PatSnap simple patent families follow the European Patent Office DOCDB definition [11]. Application publications, grant publications, Patent Cooperation Treaty (PCT) publications, and national or regional phase records sharing the same simple patent family ID were counted as 1 family. Divisional or continuation applications were grouped with the parent application when they shared the same family ID and counted separately when the IDs differed. PatSnap-assigned simple patent family IDs were used directly; families were not manually merged or split on the basis of application numbers or priority information.

The simple patent family was the unit of analysis. The earliest priority year and priority country were used to describe the origin of each family. Non-English records were not excluded. English titles and abstracts supplied by PatSnap were used where available; records with missing or insufficient English text underwent manual review.

All members of each simple patent family were reviewed by patent type. Families containing at least 1 invention patent application or grant, including mixed families, were retained and counted once, whereas utility-model-only families were excluded. Chinese utility models undergo preliminary examination, whereas invention patents undergo substantive examination.

### Patent Screening

Two reviewers independently screened the patent families, first by title and abstract and then by the relevant independent claims. When eligibility remained uncertain, the full text and other family members were examined. Disagreements were resolved through discussion. The numbers of included and excluded families and the reasons for exclusion are presented in the patent screening flow diagram.

A patent family was included if its claims covered an IABP or its balloon, catheter, insertion or positioning components, drive or pneumatic system, triggering or timing control, monitoring or safety functions, or IABP-specific accessories. Families were excluded if IABP appeared only in the background section or a list of optional devices, or if the invention primarily concerned general-purpose balloons or catheters, other circulatory support devices, drugs, biomaterials, or clinical management.

### Rule-Based Provisional Classification and Full-Dataset Manual Adjudication

Each included patent family was assigned 1 primary technology category and 1 primary functional category. The technology categories were: (1) balloon components, including balloon geometry, membrane materials, dimensions, and related structural designs; (2) catheters and accessories, including catheters, guidewires, sheaths, insertion or positioning devices, tubing, connectors, and valves; (3) counterpulsation timing and control, including drive units, pneumatic circuits, electrocardiogram (ECG)-based or pressure-based triggering, feedback control, and balloon inflation and deflation timing; and (4) other.

The functional categories were: (1) hemodynamic and therapeutic performance, including improvements in diastolic augmentation, coronary perfusion, blood flow, cardiac output, ventricular unloading, or myocardial oxygen demand; (2) control and measurement accuracy, including improvements in signal detection, positioning, triggering, synchronization, timing, calibration, or control precision; (3) safety and reliability, including leak prevention, balloon rupture prevention, alarms, fault detection, secure fixation, and stable operation; (4) reduction of clinical risks and complications, including reductions in thrombosis, bleeding, limb ischemia, infection, embolism, hemolysis, or vascular injury; (5) operational efficiency and ease of use, including structural simplification, reductions in device size or component number, and easier insertion, connection, replacement, maintenance, or operation; and (6) other, including testing, training, and performance evaluation. The operational definitions of the technology and functional categories are provided above. Table 1 summarizes the principal IPC classifications relevant to the 3 main technology categories.

**Table 1.** Principal International Patent Classification (IPC) classifications relevant to the technology categories of intra-aortic balloon pump (IABP) patents.

Technological domains and main IPC classification	Description of classification
Balloon components	
A61M60/139	Blood pumps positioned inside the aorta, including IABP systems
A61M60/295	Balloon pumps used for circulatory assistance
A61M60/841	Constructional details of circulatory assist balloon pumps
A61M60/843	Balloon geometry, structural configuration, and materials
A61M1/10	Historical classification for blood pumps and mechanical circulatory assistance devices
Catheters and accessories	
A61M25/00	Catheters and hollow probes
A61M25/01	Catheter introduction, guidance, advancement, placement, or fixation
A61M25/10	Balloon catheters and related structural arrangements
A61L29/00	Materials for catheters and catheter surface coatings
A61M39/00	Medical tubing, connectors, couplings, branch units, and valves
A61M60/865	Devices for guiding or inserting pumps into the patient’s body
A61M60/867	Pump deployment and position detection arrangements
Counterpulsation timing and control	
A61B5/0215	Invasive measurement of pressure in the heart or blood vessels
A61B5/318	Heart-related electrical monitoring, including electrocardiography
A61M60/497	Drive mechanisms for circulatory assist balloon pumps
A61M60/508	Electronic control and feedback regulation of blood pumps
A61M60/515	Pump regulation using real-time patient data
A61M60/531	Pump regulation using blood pressure signals
A61M60/569	Pump operation synchronized with the native heartbeat

In R version 4.4.1, titles and abstracts were standardized and classified using a prespecified keyword dictionary, regular expressions, and priority and conflict resolution rules. Each family received 1 provisional rule-based technology label and 1 provisional functional label. The rule-based assignments were used only as provisional labels and did not determine the final classifications.

Following provisional rule-based classification, 2 reviewers independently reassessed all included patent families and assigned 1 technology category and 1 functional category to each family. The reviewers were blinded to the provisional rule-based labels and to each other’s assessments. Classification was based on the title, abstract, and relevant independent claims; full texts and other family members were examined when necessary. Disagreements were resolved through discussion to establish consensus labels. The consensus human-assigned labels, rather than unreviewed rule-based labels, were used in all final analyses [12].

### Data Analysis

Data processing, statistical summaries, and visualization were performed in R version 4.4.1 using the *readxl*, *dplyr*, *stringr*, *tidyr*, *ggplot2*, *ggrepel*, *cowplot*, *ragg*, and *irr* packages. All counts were based on simple patent families.

Temporal and geographical trends were analyzed using the earliest priority year and earliest priority country or region of each simple patent family. Each family was assigned to 1 geographical category only: China, the United States, Japan, Europe, or other countries and regions. Europe included the European Patent Office. Year-region combinations with no records were assigned a value of zero.

On the basis of changes in annual filing volume, technological development was descriptively divided into an embryonic stage (1968-1985), a period of steady development (1986-2012), and a period of rapid development (2013-2026). These periods were used only to describe the results and were not derived from clustering or statistical modeling.

Applicant names were based on the standardized names provided by PatSnap. Obvious name variants belonging to the same legal entity were consolidated after manual verification, whereas parent companies and subsidiaries were generally counted separately. Full counting was used for coapplications: each patent family was counted once for every listed applicant. The 10 applicants with the largest numbers of patent families were reported.

The technology function matrix was constructed by cross-tabulating the final technology and functional categories. The number of patent families in each cell was displayed as a heatmap. No regression analyses or hypothesis tests were performed.

### Ethical Considerations

Not applicable. This study was based on patent data and patentometric analysis and did not involve human participants, human tissues, animals, or identifiable personal data.

## Results

### Overview

The patent identification and screening process is summarized in Figure 2. The initial search of the PatSnap Global Patent Database identified 2139 records. After 41 (1.9%) duplicate publication records were removed, 2098 (98.1%) records were screened. Of these, 766 (36.5%) families were excluded during relevance screening, leaving 1332 (63.5%) families. A subsequent family-level patent-type review excluded 97 utility-model-only families, resulting in 1235 (92.7%) simple patent families for the final analysis.

**Figure 2.**
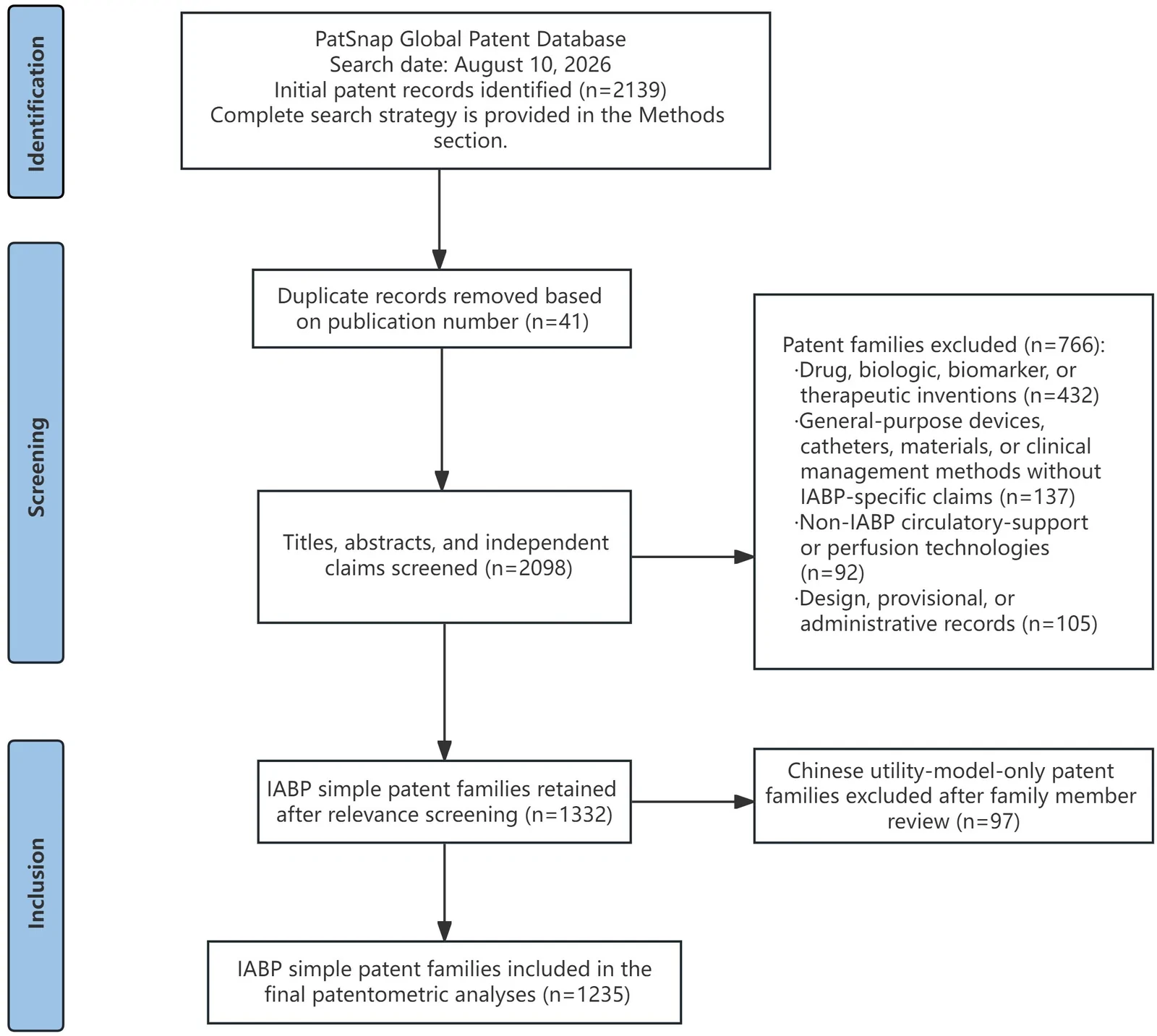
Flow diagram of the identification, screening, exclusion, and inclusion of intra-aortic balloon pump (IABP) patent families.

Before consensus, the 2 reviewers independently classified all 1235 patent families. Observed agreement was 84.8% for the technology classification (unweighted Cohen κ=0.77) and 89.2% for the functional classification (κ=0.87). These values represent agreement between the reviewers before discussion. Disagreements were resolved through discussion, and all subsequent analyses used the consensus labels.

### Analysis of the Technological Life Cycle of IABP-Related Patents

The technological life cycle of patents was divided into 3 stages: an embryonic stage, a steady development stage, and a rapid development stage. Figure 3 illustrates the annual trends in simple patent families in the IABP technology field. In 1968, Medical Research and Development (MEDRAD) filed its first patent related to IABP, marking the first documented attempt to place a balloon within the aorta for counterpulsation support. On the basis of changes in annual patenting activity, the technological evolution of IABP can be broadly divided into 3 stages.

**Figure 3.**
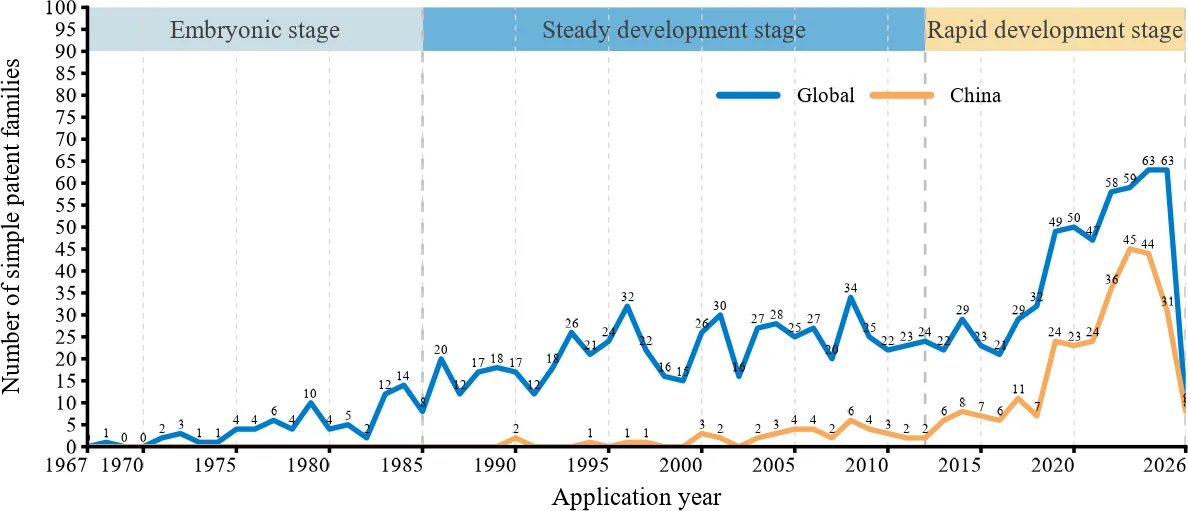
Annual number of simple patent families and technological life cycle of intra-aortic balloon pump.

First, during the embryonic stage (1968-1985), patent activity remained extremely limited, with fewer than 15 applications filed annually, indicating that the technology was still in a phase dominated by proof-of-concept and preliminary exploratory development. Second, during the steady development stage (1986-2012), the number of simple patent families began to increase gradually, with annual filings generally ranging from 10 to 40, suggesting that the technology was progressively moving beyond early experimental validation toward clinical translation. Finally, during the rapid development stage (2013-2026), the number of simple patent families increased sharply, and annual filings exceeded 50 after 2018, indicating that IABP technology entered a new period of intensified innovation activity. In China, IABP-related simple patent families first appeared in 1986. Annual family counts increased rapidly after 2017, and 55.2% of Chinese families had earliest priority dates within the most recent 5-year period, making China a major contributor to the recent increase in global IABP patenting activity.

### Analysis of the Geographical Distribution of IABP Simple Patent Families

The geographical distribution of simple patent families was analyzed according to their earliest priority country or region. As shown in Figure 4, and Multimedia Appendix 1, the United States, China, Japan, and Europe together accounted for 89.1% (1100/1235) of the included families.

**Figure 4.**
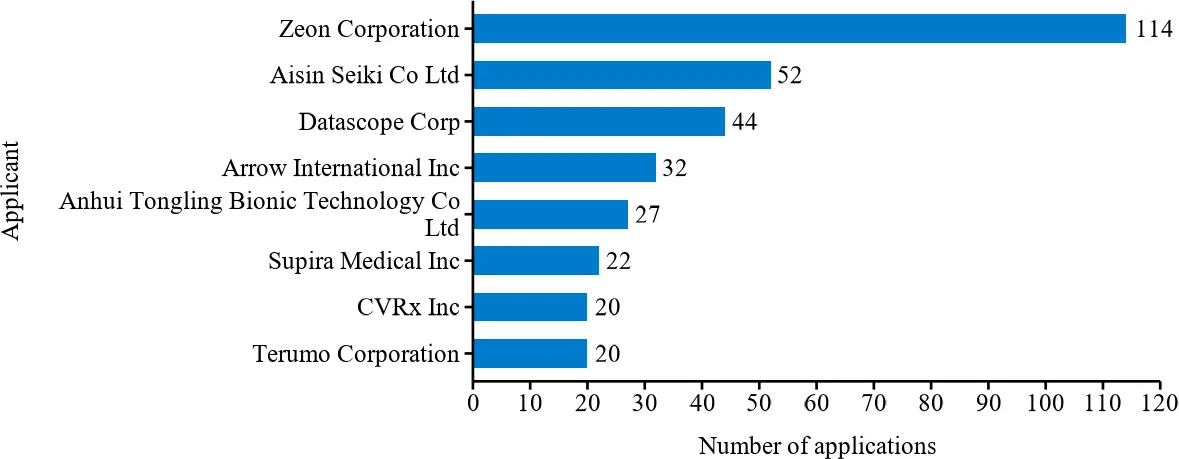
Ranking of major global applicants for intra-aortic balloon pump patents.

The United States, the place of origin of IABP technology, accounted for 40.7% (503/1235) of all simple patent families worldwide, with major applicants including established industry leaders such as Datascope Corporation and Teleflex, including its Arrow business. This distribution reflects the long-standing technological and commercial advantages of incumbent enterprises in this field.

China contributed 326 simple patent families, accounting for 26.4% of the global total. Of these, 55.2% (180/326)had earliest priority dates within the most recent 5-year period, highlighting a pronounced acceleration in innovation activity. The profile of major applicants in China is notably diverse, encompassing enterprises such as Anhui Tongling Bionic Technology Co Ltd; clinical institutions including Fuwai Hospital of the Chinese Academy of Medical Sciences and West China Hospital of Sichuan University; and innovation-oriented companies such as Shanghai NewMed Medical, thereby reflecting a collaborative pattern of innovation spanning industry, academia, and clinical practice [13].

Japan accounted for 17.6% (217/1235) of global simple patent families, with filings concentrated primarily among companies with established strengths in materials science, such as Zeon Corporation, suggesting a comparatively deep accumulation of expertise in balloon-related material technologies.

Although Europe accounted for a relatively small proportion of simple patent families (4.3% (53/1235) of the global total), it remains an important target market and continues to hold strategic value in the international patent landscape of IABP technology.

### Analysis of Technological Directions and Research Hotspots in Research and Development Related to IABP Patents

The technology function matrix showed that patent activity was concentrated on hemodynamic and therapeutic performance, control and measurement accuracy, and operational efficiency and usability (Figure 5). These functions were mainly associated with balloon components and catheters and accessories, whereas fewer patents addressed safety, reliability, and clinical risk reduction.

On the basis of their principal inventive contributions, the patents were organized into 3 core technological directions: balloon components, catheters and accessories, and counterpulsation control (Figure 6).

**Figure 5.**
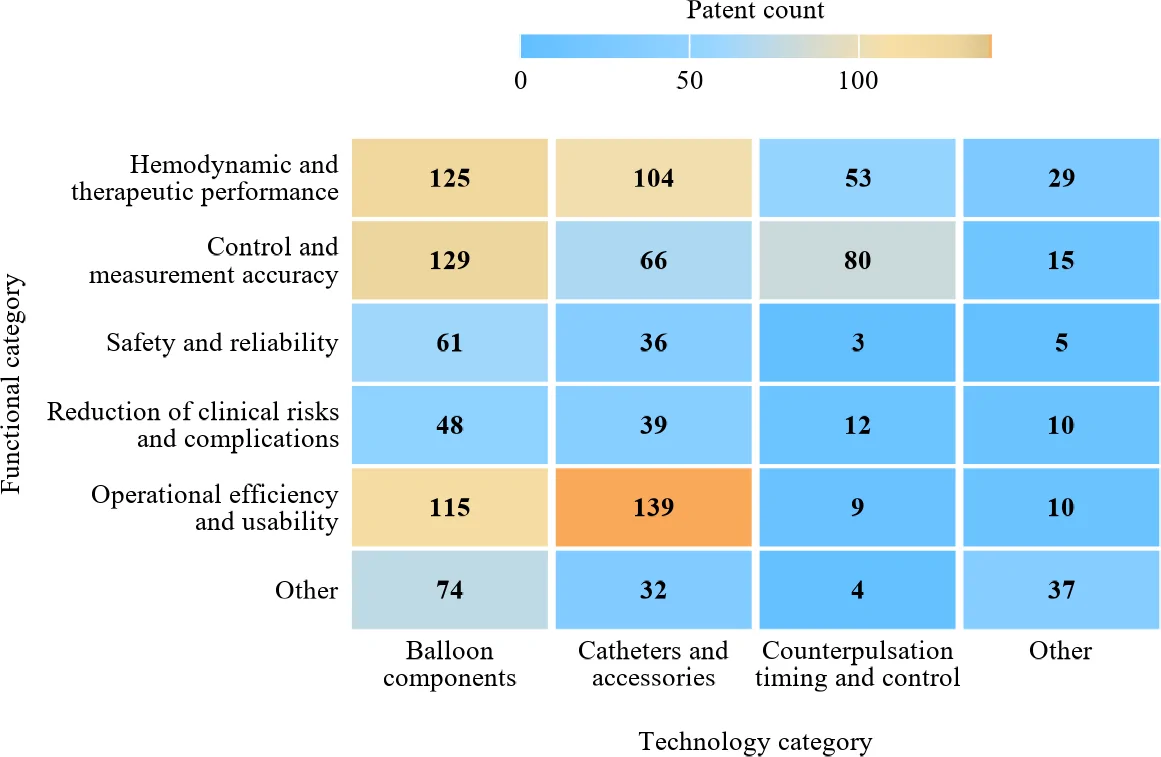
Technology function matrix of the 1235 intra-aortic balloon pump patent families.

**Figure 6.**
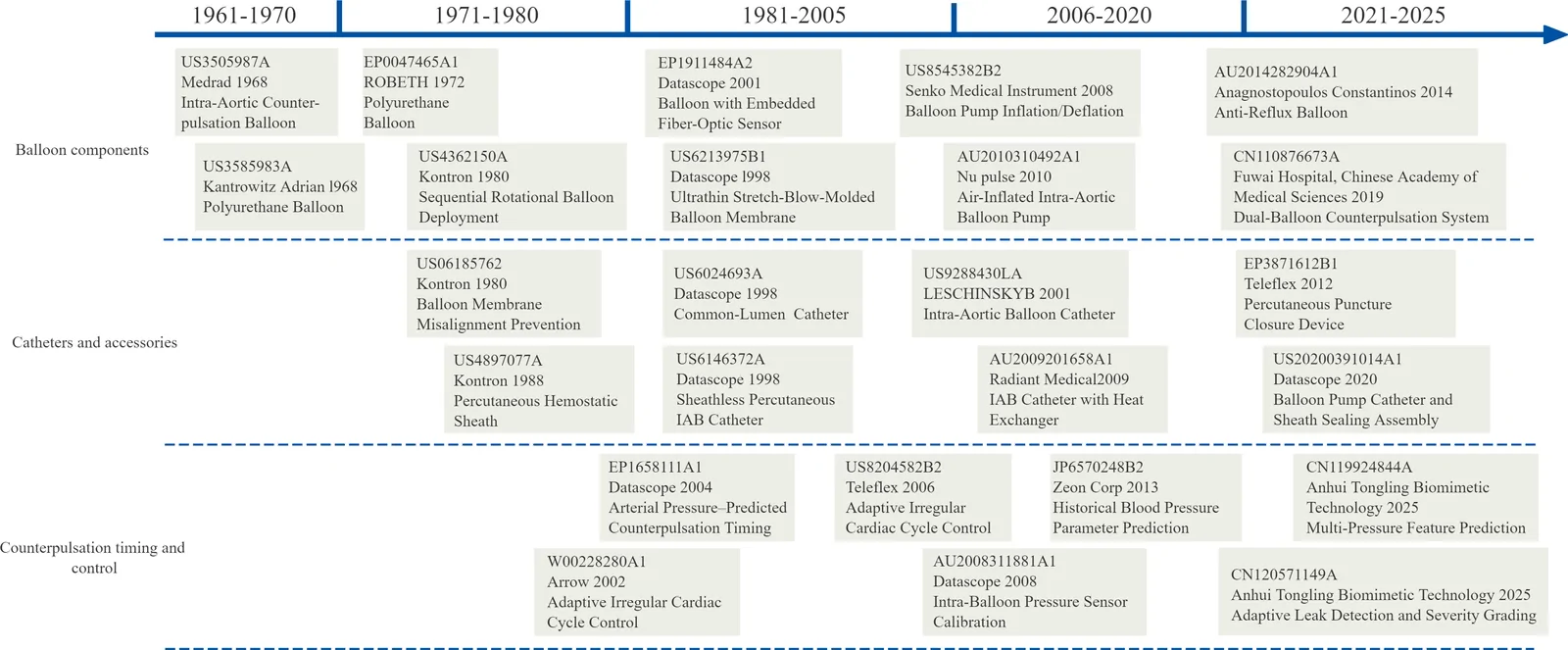
Technology road map of intra-aortic balloon pump (IABP). IAB: intra-aortic balloon.

### Balloon Components

Patents related to balloon components primarily addressed folding and compression, positioning, and inflation performance.

Prefolding, controlled wrapping around a central support member, removable stylet delivery, and flexible energy-storing supports facilitated percutaneous insertion. Reduced balloon membrane thickness further decreased the wrapped profile and enabled use through smaller-caliber access routes, as shown in US4362150A, US4402307A, and US6213975B1.

Positioning-related patents addressed balloon length selection for patients of different body sizes, positional stability after implantation, and reliable intraoperative localization [14].

Later patents optimized membrane thickness and regional wall thickness distribution to improve inflation-deflation response and durability [15], including JP2022093711A and US5484385A. CN110876673B and CN115282469B proposed dual-balloon configurations and differentiated inflation-deflation strategies for directional or adjustable circulatory support. These configurations remain patent stage concepts rather than established clinical designs.

### Catheters and Accessories

Patents concerning catheters and accessories focused on reducing device profile while maintaining delivery performance, structural strength, and gas transfer efficiency.

The main approaches included compact folding structures and guidewire-assisted delivery to reduce insertion trauma; kink-resistant shafts and thin-walled, high-strength materials to preserve gas lumen area while reducing external diameter; and co-lumen designs to improve guidewire tracking and gas transfer. Representative patents included US4362150A and US6024693A.

Alternative insertion and vascular access configurations were also described. US6146372A applied percutaneous sheathless insertion to pediatric IABP, whereas US7468050B1 proposed upper-body arterial access for long-term ambulatory support.

### Precise Control of Counterpulsation

Patents related to counterpulsation control focused mainly on sensing and control algorithms.

Sensing-related patents covered electrocardiogram-based R-wave detection, arterial pressure–based dicrotic notch detection, and catheter-tip pressure sensing. US20020072679A1 incorporated a fiber-optic pressure sensor into an IABP catheter for arterial pressure measurement and waveform landmark identification.

Control algorithm patents included fixed timing and adaptive regulation. Two recent patents from Anhui Tongling Bionic Technology Co Ltd addressed electrocardiographic morphology-guided feature point detection and pressure-based adaptive control, respectively: CN119924844A and CN120571149A [16,17]. Patent disclosure alone does not demonstrate clinical adoption or clinical influence.

A smaller group of patents concerned dedicated testing platforms, including mock circulatory loops for evaluating pressure augmentation, inflation-deflation timing, catheter positioning, triggering errors, and device failure modes under reproducible conditions [18].

## Discussion

This study integrated patentometric analysis with clinical evidence and commercial products to examine the evolution of IABP technology and its alignment with clinical needs. IABP patent activity was concentrated in the United States, China, Japan, and Europe. Earlier patent families originated mainly from the United States, Japan, and Europe, whereas the number and proportion of families with China as the earliest priority country increased markedly in recent years. However, this increase reflects patenting activity rather than equivalent growth in clinical adoption, commercialization, market share, or technological influence.

IABP innovation gradually shifted from balloon and catheter optimization toward sensing, control, and system integration. Early patents focused mainly on balloon structures, catheter design, and inflation-deflation mechanisms, whereas later inventions increasingly addressed miniaturization, positioning, pressure sensing, trigger detection, adaptive timing, fault monitoring, and testing platforms. Overall, 3 major development pathways were identified: balloon catheter structural optimization, sensing and adaptive control, and testing and validation platforms.

First, optimization of balloon catheter structures remains fundamental to improving counterpulsation performance, insertion safety, and device durability. Balloon design must accommodate anatomical variation while maintaining positional stability and reliable intraoperative localization, because malposition may result from either improper proximal placement or a mismatch between balloon length and patient anatomy [14]. The inflated balloon diameter should generally remain less than approximately 90% of the aortic diameter to balance hemodynamic support against the risk of vascular obstruction [15]. Related patents have sought to improve counterpulsation performance, vascular compatibility, and durability through modifications to membrane thickness, regional wall thickness distribution, and inflation-deflation strategies.

Catheter caliber, folding structure, and access route also influence the safety and clinical applicability of IABP. Catheter size has decreased from 12 to 15 Fr in early systems to 7 to 8 Fr, thereby reducing insertion trauma and the risk of vascular complications [19]. Sheathless insertion, guidewire compatibility, and axillary or subclavian access have further expanded the potential use of IABP for prolonged support and as bridge therapy [20-22]. Commercial products reflect similar priorities. The Datascope and Getinge Linear and Mega catheters (Datascope Corp) emphasize low-profile design and larger balloon volume, respectively, whereas the Teleflex and Arrow UltraFlex (Teleflex Incorporated), Ultra 8 (Teleflex Incorporated), and RediGuard (Teleflex Incorporated) catheters incorporate reinforced shafts, sheathless insertion, or abrasion-resistant balloon membranes [23,24]. These products were included only to provide clinical and industrial context; patent counts should not be used to infer market share, clinical uptake, or clinical influence.

Second, sensing and adaptive timing control have become major directions for improving counterpulsation accuracy. The balloon should inflate after aortic valve closure and deflate before left ventricular ejection. Incorrect timing may increase afterload, reduce ventricular unloading, and raise myocardial oxygen demand [25]. Conventional electrocardiographic and pressure-based triggering is vulnerable to arrhythmias, noise, and waveform distortion, whereas fiber-optic sensing, multisource signal fusion, and adaptive algorithms may improve the reliability of trigger detection and timing control [26]. Products such as Cardiosave (Datascope Corp), Sensation (Datascope Corp), FiberOptix, and AC3 Optimus incorporate fiber-optic pressure sensing, automatic calibration, or adaptive timing functions [27,28]. Nevertheless, the optimal synchronization strategy across different counterpulsation ratios, access routes, shock phenotypes, and irregular rhythms remains uncertain [28,29].

Finally, dedicated testing and validation platforms provide a basis for systematic evaluation of IABP devices and control strategies. Mock circulatory loops and other bench systems can assess pressure augmentation, inflation-deflation timing, catheter position, triggering errors, and device failure under reproducible conditions [18]. They may also support device comparison, algorithm validation, and clinical training. Their potential applications extend beyond basic performance testing to complex scenario simulation, risk identification, and system-level validation.

The technology function matrix further showed that patent activity was concentrated mainly on hemodynamic performance, control and measurement accuracy, and operational efficiency, whereas relatively few inventions directly addressed safety, reliability, or clinical risk reduction. At the same time, bleeding, limb ischemia, stroke, catheter displacement, and device malfunction remain unresolved concerns [30]. This gap suggests that current patenting priorities do not fully reflect unmet clinical needs and that future innovation should place greater emphasis on complication prevention and device reliability [31].

Despite advances in device structure, control, and validation, the hemodynamic support provided by IABP remains modest, and its clinical value depends strongly on patient selection and treatment context. Current evidence has not shown that routine IABP use improves survival in acute myocardial infarction complicated by cardiogenic shock [32]. A recent meta-analysis of randomized trials in heart failure–related cardiogenic shock suggested possible benefit in patients with more severe shock stages, but the evidence remains limited [33]. Observational studies have produced context-dependent findings: 1 study found no reduction in short- or long-term mortality and more short-term adverse events [34], whereas selected cohorts undergoing extracorporeal cardiopulmonary resuscitation (ECPR) or percutaneous coronary intervention (PCI) with mechanical circulatory support showed associations with 30-day survival or lower resource use and complication rates relative to comparator groups [35,36]. Because these observational comparisons remain susceptible to residual confounding, IABP should be evaluated within clearly defined clinical populations rather than assumed to have a uniform effect.

By integrating temporal trends, geographical distribution, technological classification, and the technology function matrix with clinical evidence and commercially available products, this study identified the transition of IABP technology from structural optimization toward sensing, control, and system-level validation. It also revealed a gap between patenting priorities and clinical safety needs. This framework may inform future technology planning, device design, and the selection of evaluation end points, while helping to determine whether emerging technologies address clinically relevant unmet needs.

On the basis of these findings, future IABP systems should combine low-profile, durable balloon catheter structures with multisource sensing, adaptive control, continuous position monitoring, early fault detection, and scenario-specific bench testing. These technologies should be assessed not only by hemodynamic measures such as pressure augmentation, coronary perfusion, and ventricular unloading but also by their safety, reliability, ease of use, and compatibility with clinical workflows.

This study has several limitations. Patent activity reflects research and development intentions rather than patent quality, commercialization, market share, clinical adoption, or therapeutic effectiveness. The analysis remained primarily descriptive and did not include patent quality indicators, citation analysis, or measures of applicant concentration. In addition, scientific literature and commercially available products were used only to provide clinical and industrial context and were not examined through a systematic review or formal market analysis.

In conclusion, IABP technology has progressed from balloon and catheter miniaturization toward integrated sensing, adaptive control, and dedicated validation platforms. The geographical origins of IABP patent families were concentrated in a small number of countries and regions. Both the number and proportion of simple patent families with China as the earliest priority country increased markedly in recent years. However, this pattern reflects changes in the geographical origin of patenting activity rather than leadership in clinical use, commercialization, or market share. Future development and evaluation of IABP technology should move beyond hemodynamic support alone and place greater emphasis on safety, reliability, and alignment with specific clinical needs.

## Supplementary material

10.2196/98093 Multimedia Appendix 1 Annual trends in intra-aortic balloon pump simple patent families by earliest priority country or region.

## Abbreviations

ECG: electrocardiogram
ECPR: extracorporeal cardiopulmonary resuscitation
IABP: intra-aortic balloon pump
IPC: International Patent Classification
MEDRAD: Medical Research and Development
PCI: percutaneous coronary intervention
PCT: Patent Cooperation Treaty
pMCS: percutaneous mechanical circulatory support
